# Biochemical Aspects That Lead to Abusive Use of Trimetazidine in Performance Athletes: A Mini-Review

**DOI:** 10.3390/ijms25031605

**Published:** 2024-01-28

**Authors:** Amalia Pușcaș, Ruxandra Ștefănescu, Camil-Eugen Vari, Bianca-Eugenia Ősz, Cristina Filip, Jana Karlina Bitzan, Mădălina-Georgiana Buț, Amelia Tero-Vescan

**Affiliations:** 1 Biochemistry and Chemistry of the Environmental Factors Department, Faculty of Pharmacy, George Emil Palade University of Medicine, Pharmacy, Science, and Technology of Târgu Mureș, 540142 Târgu Mureș, Romania; amalia.puscas@umfst.ro (A.P.); cristina.filip@umfst.ro (C.F.); 2 Pharmacognosy and Phytotherapy Department, Faculty of Pharmacy, George Emil Palade University of Medicine, Pharmacy, Science, and Technology of Târgu Mureș, 540142 Târgu Mureș, Romania; 3 Pharmacology and Clinical Pharmacy Department, Faculty of Pharmacy, George Emil Palade University of Medicine, Pharmacy, Science, and Technology of Târgu Mureș, 540142 Târgu Mureș, Romania; camil.vari@umfst.ro (C.-E.V.); bianca.osz@umfst.ro (B.-E.Ő.); 4 Medical Chemistry and Biochemistry Department, Faculty of Medicine in English, George Emil Palade University of Medicine, Pharmacy, Science, and Technology of Târgu Mureș, Campus Hamburg—UMCH, 22761 Hamburg, Germany; jana.rivdeneira@yahoo.com; 5 Medical Chemistry and Biochemistry Department, Faculty of Medicine in English, George Emil Palade University of Medicine, Pharmacy, Science, and Technology of Târgu Mureș, 540142 Târgu Mureș, Romania; madalina.but@umfst.ro (M.-G.B.); amelia.tero-vescan@umfst.ro (A.T.-V.)

**Keywords:** trimetazidine, doping, metabolic modulator, oxidative stress

## Abstract

Trimetazidine (TMZ), used for treating stable angina pectoris, has garnered attention in the realm of sports due to its potential performance-enhancing properties, and the World Anti-Doping Agency (WADA) has classified TMZ on the S4 list of prohibited substances since 2014. The purpose of this narrative mini-review is to emphasize the biochemical aspects underlying the abusive use of TMZ among athletes as a metabolic modulator of cardiac energy metabolism. The myocardium’s ability to adapt its energy substrate utilization between glucose and fatty acids is crucial for maintaining cardiac function under various conditions, such as rest, moderate exercise, and intense effort. TMZ acts as a partial inhibitor of fatty acid oxidation by inhibiting 3-ketoacyl-CoA thiolase (KAT), shifting energy production from long-chain fatty acids to glucose, reducing oxygen consumption, improving cardiac function, and enhancing exercise capacity. Furthermore, TMZ modulates pyruvate dehydrogenase (PDH) activity, promoting glucose oxidation while lowering lactate production, and ultimately stabilizing myocardial function. TMZs role in reducing oxidative stress is notable, as it activates antioxidant enzymes like glutathione peroxidase (GSH-Px) and superoxide dismutase (SOD). In conclusion, TMZs biochemical mechanisms make it an attractive but controversial option for athletes seeking a competitive edge.

## 1. Introductions

**Figure 1:**
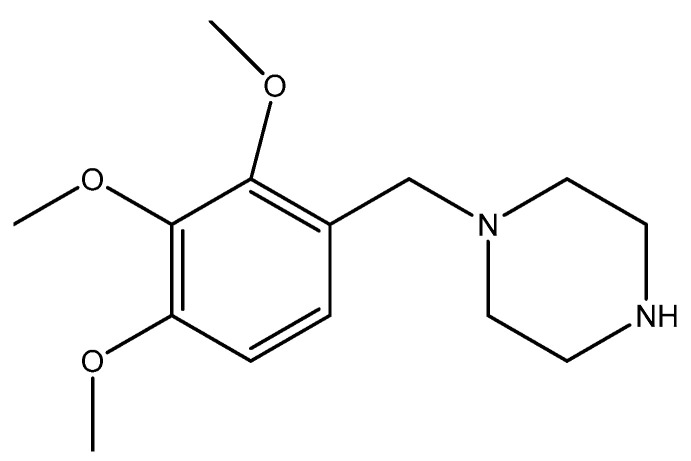
Trimetazidine structure.

Trimetazidine (TMZ) [1-(2.3.4-trimethoxybenzyl)-piperazine] is a piperazine derivative with a structure shown in Figure 1. It was introduced on the market in 1963 in France for the treatment of (stable) angina pectoris [1].

TMZ is part of the class of anti-anginal drugs with protective effects against ischemia-induced heart injury, acting as a modulator of cardiac energy metabolism between obtaining ATP from the beta-oxidation of fatty acids and glycolysis, maintaining the intracellular levels of phosphocreatine (PCr) and ATP, reducing the level of lactate and cellular acidosis, preventing calcium overload, and reducing the concentration of free radicals [2,3]. As cellular oxygen consumption in the case of glucose oxidation is lower than in the case of long-chain fatty acids, TMZ increases the survival of cardiomyocytes in ischemic conditions [3,4,5]. Other studies show a reduction in oxidative stress parameters resulting mainly from lipid peroxidation [6]. To our knowledge, there is no literature available that addresses the potential risks associated with the use of TMZ in sports, whether by professional athletes or those engaging in recreational activities. Even though TMZ has protective effects on cardiomyocytes, the principle of fair play in sports prohibits the use of substances that provide athletes with unjustifiable and direct advantages.

All these mechanisms make TMZ a compound with potential uses for improving performance in athletes. TMZ is included in the list of prohibited substances during and outside the competition of World Anti-Doping Agency (WADA) S4 hormone and metabolic modulators since 2014, together with aromatase inhibitors, anti-estrogens, selective estrogen receptor modulators (SERM), agents preventing activin receptor IIB activation, and metabolic modulators [7].

International media reports cases of athletes doped with TMZ (Chinese swimmer Sun Yang in 2014, Russian bobsledder Nadezhda Sergeeva in 2018 at the Pyeongchang Olympics, or Russian skater Kamila Valieva in 2022 at the Beijing Winter Olympics). The literature focus was more on detection methods from biological samples but was incomplete regarding the pharmacological reasons behind this abusive use. To compensate for this, the aim of this mini-review is to highlight the most important biochemical mechanisms that could bring unfair benefits to athletes doped with TMZ during or out of competition [8,9].

## 2. Methodology

**Figure 2:**
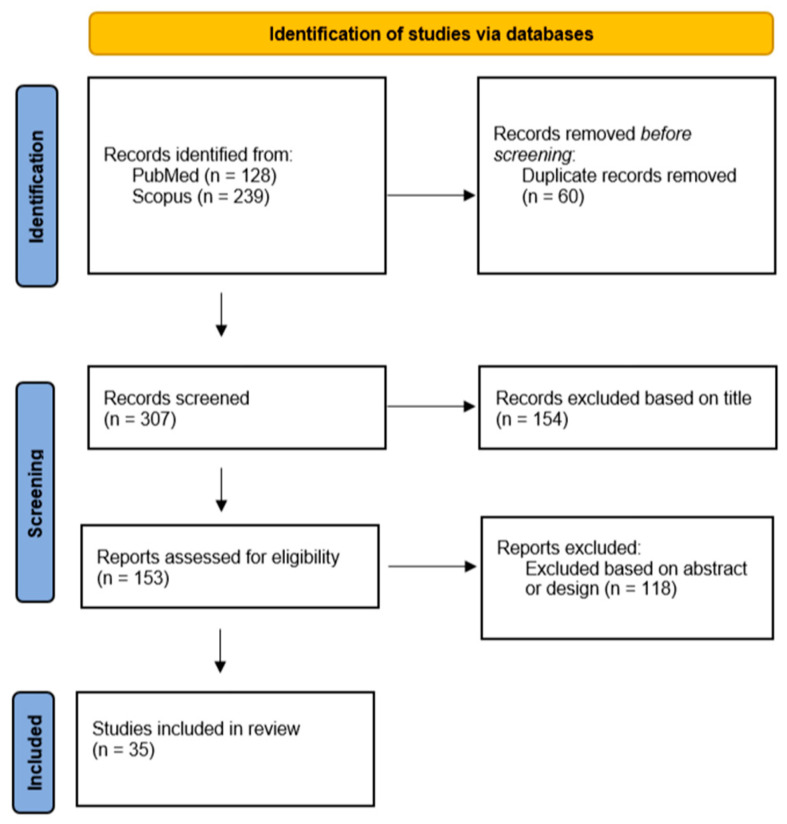
Flow diagram of the literature review process; n = number of articles.

For this narrative review, a systematic search on Scopus and PubMed was performed using combinations of the following keywords: ‘‘trimetazidine’’, ‘‘doping’’, ‘‘oxidative stress’’, and ‘‘metabolic modulator’’. EndNote 9 software was used to collect the articles, and after removing duplicates, screening the title, abstract, and study design, articles were retained for the narrative review (Figure 2). Non-English articles as well as gray literature were excluded. Additionally, articles unrelated to the biochemical aspects of trimetazidine or its use in doping were excluded based on their title, abstract, and study design. Review articles as well as original articles of research, both preclinical and clinical studies, written in English, were included, with an emphasis on the latest published articles guided by the experience of the authors. This approach aimed to ensure an up-to-date synthesis of the literature, with a specific focus on the biochemical mechanisms that explain the use of trimetazidine as a metabolic modulator.

## 3. Energy Metabolism of the Myocardium

**Figure 3:**
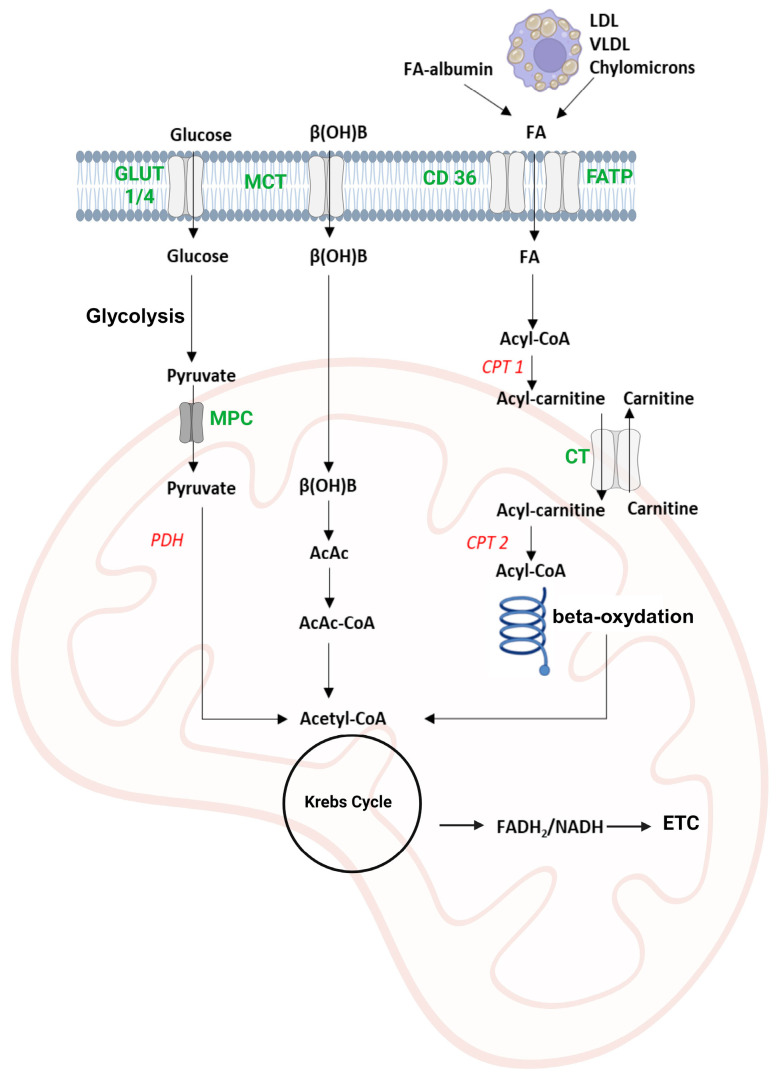
Energy metabolism of cardiomyocytes. The figure describes the uptake of the most important energy substrates in the myocardium: carbohydrates (glucose), fatty acids, and ketone bodies (beta-hydroxybutyrate). Glucose is processed by glycolysis in the cytoplasm, followed by the entry of pyruvate into the mitochondria, where it is oxidatively decarboxylated with the formation of acetyl-CoA. The fatty acids are activated into acyl-CoA and transferred to the mitochondria linked to carnitine. In mitochondria, the process of beta-oxidation will generate more molecules of acetyl-CoA, and the ketone bodies represented by beta-hydroxybutyrate and acetoacetate are metabolized by ketolysis to acetyl-CoA. The acetyl-CoA formed is metabolized in the Krebs cycle with the formation of reduced coenzymes (NADH and FADH_2_) that will be oxidized in the electron transport chain with energy formation, a part of which is stored in the form of ATP. (β(OH)B–β-hydroxybutyrate, FA–fatty acid, MCT–monocarboxylic acid transporter, CD 36–cluster of differentiation 36, FATP–fatty acid transport protein, GLUT–glucose transporter, MPC–mitochondrial pyruvate carrier, CPT 1 and 2–carnitine palmitoyl transferase 1 and 2, CT–carnitine translocase, PDH–pyruvate dehydrogenase, AcAc–acetoacetate, ETC–electron transfer chain). The text in red letters signifies enzymes and in green letters signifies transporters.

Maintaining the contractile function of the myocardium consumes significant amounts of ATP, mainly obtained from the combined use of beta-oxidation of fatty acids, metabolism of glucose, ketone bodies, and amino acids, as presented in Figure 3 [10].

### 3.1. Beta-Oxidation of Fatty Acids

The fatty acids used for energy purposes by the myocardium originate from adipose tissue following the process of lipolysis of triglycerides by hormone-sensitive lipase or from lipoprotein fractions such as chylomicrons or very low-density lipoproteins (VLDL) catalyzed by lipoprotein lipase [11]. They are transported in the blood in free form or bound to plasma albumin for uptake by cardiomyocytes via transporters such as cluster of differentiation 36 (CD 36) and fatty acid transport proteins (FATP) [12]. In the cytoplasm, fatty acids are activated by fatty acyl-CoA synthetase through an ATP-dependent reaction with coenzyme A, leading to the formation of acyl-CoA, which can be bound to glycerol to form deposit triglycerides, used for the synthesis of membrane phospholipids, or transported in the mitochondria to be oxidized for energy purposes [13,14]. Since the mitochondrial double membrane layer is impermeable to acyl-CoA, transport is carried out with the help of the carnitine transporter and 3 enzymes: carnitine palmitoyl transferase 1 (CPT-1) which catalyzes the esterification of fatty acids with carnitine, a translocase that transfers acyl-carnitine in the mitochondria, and CPT-2 located in the inner membrane of the mitochondria that releases the acyl residue from carnitine and binds it to coenzyme A [15]. The beta-oxidation of fatty acids includes four reactions: an oxidation reaction catalyzed by acyl-CoA dehydrogenase with the formation of NADH and an unsaturated compound, enoyl-CoA; a hydration with the formation of 3-hydroxyacyl-CoA; an oxidation catalyzed by 3-hydroxyacyl-CoA dehydrogenase, FAD-dependent, in which a 3-ketoacyl-CoA is formed; and a thiolytic cleavage by 3-ketoacyl-CoA thiolase (KAT) with the formation of acetyl-CoA and an acyl-CoA residue with (n-2) carbon atoms [16]. Acetyl-CoA will be oxidized in the Krebs Cycle with the formation of CO_2_ and reduced coenzymes (NADH and FADH_2_) that will supply electrons to the electron transport chain (ETC) with energy formation, stored in the form of ATP through the oxidative phosphorylation process [17].

### 3.2. Carbohydrate Metabolism

Glucose can enter the cardiac myocytes with the help of insulin-dependent GLUT-4 and insulin-independent GLUT-1 transporters and is phosphorylated with the formation of glucose-6-phosphate [18]. Glycolysis takes place in the cytoplasm with the formation of a minimal amount of energy: 2 ATP/mol of glucose and 2 molecules of pyruvate. Pyruvate can be transformed into lactate, or in the presence of oxygen, it is converted to acetyl-CoA in the mitochondrial matrix by oxidative decarboxylation via the pyruvate dehydrogenase (PDH) pathway [19]. PDH is controlled covalently by PDH kinase, by the energy status of the cell, and by the NADH/NAD^+^ and acetyl-CoA/CoA-SH ratios >1, which have an activating effect on PDH kinase and an inhibitory effect on PDH. The myocardium contains glycogen deposits, with a still-debated purpose, but it is assumed that it has an energy-buffer role, involved in the fine regulation of the energy flow of glucose in the heart, as well as an important role in determining myocardial metabolic health [20,21].

### 3.3. Ketone Bodies Metabolism

Acetoacetate and beta-hydroxybutyrate are uptaken in cardiomyocytes by passive diffusion or via monocarboxylic acid transporters. Beta-hydroxybutyrate is oxidized to acetoacetate, which will be esterified with coenzyme A in a reaction catalyzed by succinyl CoA 3-ketoacid-CoA transferase (SCOT). The formed acetoacetyl-CoA is cleaved, resulting in the formation of two molecules of acetyl-CoA, which will enter the Krebs cycle [22].

## 4. Biochemical Changes during Exercise

The myocardium can permanently switch between the use of glucose or fatty acids for energy purposes; this competition has been the subject of many studies since 1963 when the “Randle Cycle” was first mentioned [23,24]. Cardiac cells present many mitochondria in which the synthesis of ATP from different substrates takes place, so, during an acute, intense effort, carbohydrates will be the main source of energy, while in resting conditions, fatty acids will be used [21].

During fasting, lipolysis is initiated with the release of fatty acids, which can, ultimately, generate ketone bodies in the liver. Fatty acids and ketone bodies have a glucose conservation effect, which is extremely important, especially for maintaining brain function during periods of starvation. The inhibitory effect on carbohydrate metabolism is directed especially to myocardial, hepatic, and beta-pancreatic cell PDH, which thus leads to the accumulation of lactate and pyruvate, both important precursors for gluconeogenesis [25].

In cases of physical effort or after a meal rich in lipids, the increased concentration of free fatty acids and ketone bodies in the blood has an inhibitory effect on glycolysis, with glucose being redirected to form glycogen deposits, which explains the rapid replenishment of glycogen deposits post-physical effort [26]. This metabolic switch is due to the inhibitory effect on PDH through the ratios NADH/NAD^+^ and Acetyl-CoA/CoA-SH >1, but also through the direct, allosteric inhibitory action of citrate formed in excess in the mitochondria on the key enzymes of glycolysis, 6-phosphofructo-1 kinase (PFK-1) and PFK-2. PFK-2 catalyzes the formation of fructose 2,6-bisphosphate (F-2,6-P_2_), which is an activator of PFK-1. Therefore, the accumulation of citrate in the cytoplasm has a double inhibitory effect on glycolysis: allosteric and by decreasing the concentration of F-2,6-P_2_ [19,27].

In conditions of metabolic stress such as acute, high-intensity cardiac effort, the inhibitory effect of beta-oxidation of fatty acids on glycolysis is removed and AMP-activated protein kinase (AMPK) is activated, with an essential role in maintaining caloric homeostasis [28]. AMPK is activated during metabolic stress induced either by glucose or oxygen deprivation or by increased cellular energy demands during intense muscular effort, both of which are characterized by an AMP/ATP ratio >1. It determines the activation of ATP-generating catabolic processes (beta-oxidation of fatty acids or glycolysis) and the inactivation of energy-consuming anabolic processes. AMPK can determine in an insulin-independent way the uptake of glucose into the ischemic heart cell, but it can also directly modulate by phosphorylating key enzymes in carbohydrate, lipid, or protein metabolism, such as the inactivation of acetyl-CoA carboxylase (ACC), the enzyme that catalyzes the key step in the de novo synthesis of fatty acids, the transformation of acetyl-CoA into malonyl-CoA, or the activation of PFK-2 and implicitly of glycolysis, but also of the Akt substrate 160, a protein that determines the incorporation of GLUT-4 transporters for glucose in the cell membrane [29,30].

Metabolic stress can also be induced by stimulating the myocardium with epinephrine. This will cause increased glycolysis via the second messenger, cyclic adenosine monophosphate (cAMP), and intramitochondrial [Ca^2+^]. cAMP determines the activation of cyclic AMP-dependent protein kinase (PKA) and thus, through phosphorylation, decreases the activity of ACC, favors glucose uptake, activates glycogen phosphorylase, and PDH. In these conditions, glucose is preferentially used as an energy substrate to support the cardiac effort induced by epinephrine [31].

## 5. TMZ Mechanism of Action

TMZ is an antianginal drug that belongs to the group of partial inhibitors of fatty acid oxidation (PFox inhibitors). Even if the mechanism of action is not fully elucidated, it has demonstrated cardioprotective effects similar to nifedipine or propranolol in stable pectoral angina by modulating cardiac energy metabolism [32]. TMZ is a 3-ketoacyl CoA thiolase (KAT) inhibitor, the enzyme involved in the process of mitochondrial beta-oxidation of fatty acids, thus reducing the use of long-chain fatty acids as an energy substrate in the myocardium, leading to a better use of carbohydrates through the process of glycolysis and thereby decreasing proton and Na^+^ accumulation [33]. Even if the beta-oxidation of fatty acids produces a greater amount of energy than glycolysis, it still increases the oxygen consumption of the cell [34]. Paradoxically, in conditions of hypoxia, when both beta-oxidation and aerobic use of glucose are modified, in the cells the use of fatty acids for energy purposes is preferred, which will exacerbate oxygen consumption, and the end product of glycolysis is lactate, responsible in severe cases for metabolic acidosis [35].

Another important mechanism of action of TMZ is the activation of PDH, the enzyme that couples intracytoplasmic glycolysis with pyruvate transformation in the mitochondria into acetyl-CoA through oxidative decarboxylation. Compared to the beta-oxidation process, energy obtained in this way decreases the oxygen consumption of the cell, decreases the production of [H^+^], increases the risk of acidosis, and increases the intracellular accumulation of Ca^2^+ with a stabilizing effect on the membrane [36,37].

**Figure 4:**
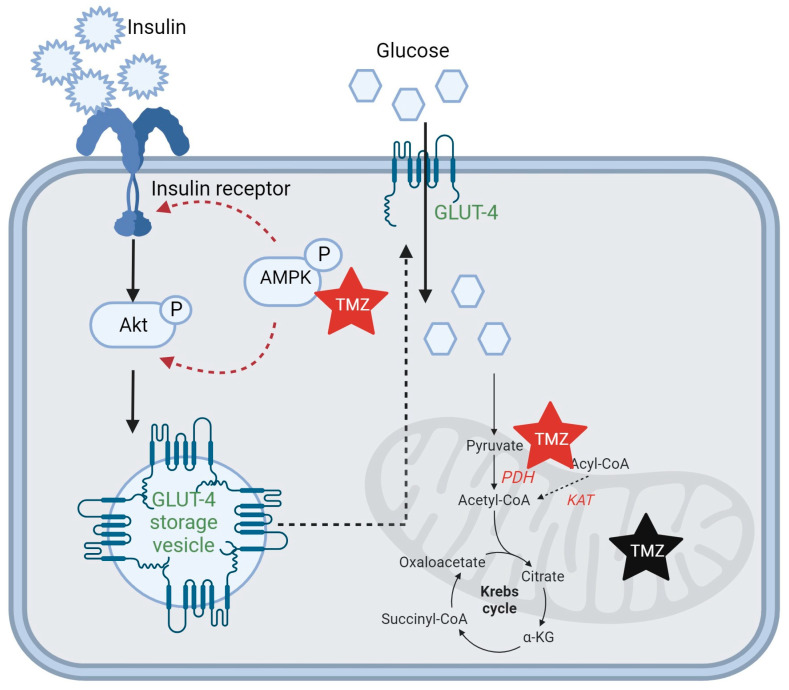
TMZ mechanism of action. TMZ is an inhibitor of KAT, decreasing fatty acid β-oxidation, and an activator of PDH, an enzyme that catalyzes the transformation of pyruvate to acetyl-CoA, making it a metabolic modulator from using fatty acids as a source of energy to glucose. TMZ activates AMPK and Akt through phosphorylation. GLUT-4 insulin-activated receptors for glucose are externalized from storage vesicles to the cell membrane. (TMZ–trimetazidine, KAT–3 ketoacyl-CoA thiolase, Akt–protein kinase B, GLUT-4-insulin-regulated glucose transporter, AMPK–AMP-activated protein kinase, PDH–pyruvate dehydrogenase). Red stars represent an activator effect and black star represents an inhibitor effect of TMZ on specific enzymes. A red dotted arrow signifies a process involving several steps activated by TMZ and a black dotted arrow signifies a process involving several steps inhibited by TMZ.

TMZ activates AMPK, the key enzyme in carbohydrate metabolism, and reduces insulin resistance in the skeletal muscle by externalizing GLUT-4 receptors in the cell membrane and favoring their expression through a positive loop with peroxisome proliferator-activated receptor β (PPARβ) [38]. Phosphorylated AMPK activates Akt through phosphorylating IRS1 at Ser794; see Figure 4 [39].

The improvement of cardiac function, especially of the systolic phase, by switching the use of energy substrates in favor of glucose was highlighted by a study on mice with cardiomyocyte-specific deficiency of PDH. In these conditions, the oxidative decarboxylation of pyruvate with the formation of acetyl-CoA is reduced, and the fatty acids through beta-oxidation will provide the energy necessary for cardiac function. The study shows that in cases of insufficient use of glucose for energy purposes, the amount of ATP available is sufficient for the diastolic function but not for the systolic one [40]. The importance of glucose in cardiac energy metabolism is very well documented in the case of type 2 diabetes mellitus, where the combined use of alternative energy substrates such as fatty acids, ketone bodies, and branched-chain amino acids is responsible for the occurrence of cardiomyocyte dysfunction and the development of diabetic cardiomyopathy [41]. However, this mechanism alone cannot explain the energy use of other substrates than glucose in diabetic patients. Studies show that almost all enzymes activated by insulin secretion are acetylated. High blood sugar levels produce insulin secretion and hyperacetylation of some enzymes, especially those involved in the beta-oxidation of fatty acids, leading to a decrease in their stability and activity [42].

## 6. TMZ and Oxidative Stress

Oxidative stress is an important characteristic of cardiovascular diseases, especially in the ischemic myocardium, but at the same time also in moderate to intense muscular effort. The most important sources of reactive oxygen species (ROS) are electron losses that can occur at the level of the ETC and nicotinamide adenine dinucleotide phosphate oxidase (NADPH oxidase/NOX) [43]. TMZ as a modulator of cardiac energy metabolism, can influence different markers of oxidative stress. In a study conducted on mice subjected to cardiac hypoxia following acute myocardial infarction, TMZ demonstrated protective effects against cardiac rupture (CR), a severe complication. This ischemic condition triggered the generation of ROS, activation of inflammatory cytokines, matrix metalloproteinases (MMPs), and collagen deposition, culminating in structural changes within the damaged myocardium. Furthermore, the disruption of extracellular matrix (ECM) proteins emerged as a crucial mechanism in the pathogenesis of CR, with MMPs playing a recognized role in regulating ECM protein turnover. The activation of MMPs has been identified as a key factor in the progression of post-myocardial infarction (CR). In the same experimental study, the group that received TMZ pretreatment exhibited a reduction in the expression of MMPs, specifically MMP-2 and MMP-9. Additionally, TMZ pretreatment demonstrated a significant decrease in both cardiac hydrogen peroxide (H_2_O_2_) generation and malondialdehyde (MDA) levels, indicating a notable antioxidative effect of TMZ [44].

In another study on rats, TMZ (30 and 60 mg/kg) administered before exhaustive exercise (prolonged swimming training) conferred cardioprotection by modulating key markers associated with oxidative stress-related injury. TMZ elevated the levels of glutathione (GSH) and enhanced the activities of superoxide dismutase (SOD) and glutathione peroxidase (GSH-Px), while reducing the level of MDA. TMZ inhibited oxidative stress and cardiomyocyte apoptosis by activating the Nrf2/HO1 ((nuclear factor (erythroid-derived 2)-like 2)/heme oxygenase 1) signaling pathway and deactivating the NF-κB (nuclear factor NF-kappa-B p65 subunit) signaling pathway. Top of Form Apoptosis was also inhibited by reducing the Bax/Bcl-2 (B-cell lymphoma 2/Bcl-2-associated X protein) ratio. Bcl-2 and Bax, both significant members of the Bcl-2 family, play crucial roles in the regulation of apoptosis. Bax functions as a proapoptotic protein, whereas Bcl-2 acts as an anti-apoptotic protein. Furthermore, the levels of cleaved caspase-3, cleaved poly (ADP-ribose) polymerase (PARP), and cytochrome c, critical indicators of apoptosis, were found to be decreased in the pretreated group. The levels of cardiac troponin I (cTnI) and N-terminal pro-brain natriuretic peptide (NT-proBNP), which serve as biomarkers for myocardial damage, were also decreased by pretreatment with TMZ, highlighting TMZs positive effects on myocardial protection [45].

Other studies show that TMZ reduces oxidative stress by directly activating enzymes such as GSH-Px and SOD, with a concomitant reduction of MDA levels [46]. TMZ reduces the level of Bax and increases the expression of caspase-3 and Bcl-2 in a concentration-dependent manner in an animal model of myocardial infarction, decreasing the risk of myocardial cell apoptosis [47].

A study on the influence of chronic treatment with 60 mg/day TMZ, divided into three doses, in patients with end-stage renal disease shows that the serum levels of MDA were significantly lower after 6 months of treatment, and the mortality rate due to cardiovascular complications was significantly reduced compared to the control group [48]. The administration of 20 mg of TMZ 3 times a day to patients with chronic cor pulmonale shows that after 3 months of treatment, the activities of plasma SOD, erythrocyte catalase (CAT), and GSH-Px (erythrocyte and plasma) were increased, and the amount of MDA and brain natriuretic peptide (BNP) was lower in the group treated with TMZ [49].

Additionally, there are reports indicating that mitochondrial permeability transition plays a crucial role in cardiomyocyte apoptosis. This phenomenon is a consequence of the opening of the mitochondrial permeability transition pore (mPTP) in the inner mitochondrial membrane, and it is stated that inhibiting the opening of mPTP could serve as an additional mechanism for providing cardioprotection. TMZ can also be considered a mitochondrial permeability transition pore (mPTP) inhibitor, thus having a cardioprotective effect during ischemia [50].

## 7. TMZ and Doping

TMZ was added to the WADA list of prohibited substances in January 2014 as a metabolic modulator due to a decrease in coronary vascular resistance, increased coronary blood flow, and cytoprotective/anti-ischemic effects in other organs [1].

The abuse of metabolic modulators such as TMZ in sports started with the similarities regarding the use of energy substrates with ischemic heart disease and heart failure, as the predominant use of anaerobic glycolysis as well as its decoupling from mitochondrial oxidative metabolism has an essential role in cardiac inefficiency (oxygen consumed per work performed) and functional impairment. The biochemical approaches proposed in this case would favor the oxidative metabolism of glucose and/or inhibit the beta-oxidation process [51].

During rest, the beta-oxidation of fatty acids is the main energy source (up to 80%), and approximately 20% of the maximum oxidative capacity of the myocardium cell is used. During low to moderate intensity effort, an increase in the uptake of glucose and lactate in the myocardium was observed, while in the case of high-intensity effort, the use of glucose decreases, the aerobic limit is reached, anaerobic metabolism is triggered, and ventricular performance declines [52].

By inhibiting the key enzyme of beta-oxidation, KAT, TMZ determines a metabolic switch between the predominant use of fatty acids and the use of glucose for energy purposes, which lowers demand for oxygen and improves left ventricular ejection fraction, cardiac diastolic function, quality of life, and exercise capacity [1]. For example, in the case of running medium distances (800–5000 m), the intensity of the effort is very high, ∼95% to 130% of VO_2_ max, and for performance athletes who present primarily Type IIa/IIx fiber morphology, glycogen is almost exclusively used for energy purposes both aerobically and anaerobically [53]. The amount of lactate produced following the anaerobic use of glucose is relatively high, up to 20 mmol/L, and the muscle pH value is low, up to 6.6. An important buffer in this case is muscle carnosine [54]. In these conditions, the activation of PDH by TMZ can have a favorable impact not only on the aerobic metabolism of the cell but also on the maintenance of muscle pH. In the case of ultra-marathon runners (distances higher than 42.2 km), food intake and the use of carbohydrates for energy purposes are essential for the body’s adaptation to training [55,56]. Glycogen, in addition to its role as an energy deposit, is also a regulator of metabolic signaling through direct or indirect mediation of the AMPK and p38 mitogen-activated protein kinase (MAPK) pathways. AMPK and p38 MAPK control the expression and activity of the peroxisome proliferator activated receptor γ coactivators (PGCs), which directly increase the mitochondrial mass in the muscles and implicitly the exercise capacity [57]. Intense sprinting requires rapid energy turnover, mainly originating from small sex-differences in anaerobic metabolism. The relative aerobic contribution becomes more important as the distance increases, in men from 20.4  ±  7.9% in the 100 m race to 41.3  ±  10.9% in the 400 m race and in women from 25.0  ±  7.4% in the 100 m race up to 44.5 ± 7.6% in the 400 m race [58].

Regarding the performance-enhancing effect of TMZ on the myocardium, a study conducted on patients with heart failure with preserved ejection fraction who were administered 20 mg of TMZ three times a day shows that by reducing beta-oxidation and favoring glucose oxidation, more moles of ATP/mol of oxygen consumed will be obtained, which improves mitochondrial function, reduces oxidative stress, and increases the value of the myocardial PCr/ATP ratio [59]. Studies conducted with 31P-magnetic resonance spectroscopy (31P-MRS) show that TMZ increases the value of the PCr/ATP ratio, a measure of myocardial energetics; the low value of this ratio is due to the imbalance between the intake and the oxygen requirement of the cell [60].

In the case of performance athletes, diet, dietary supplements, and prescribed medications are recommended by physicians specializing in sports medicine. However, for those engaging in recreational sports activities, concern arises regarding the debatable origin of TMZ as a prescription substance (black market or online sources), with risks of adulteration or overdose. Additionally, there is the potential danger of combining TMZ with other prohibited substances, which influences cardiac metabolism and poses risks to the life and health of the athlete.

## 8. Synergism and Antagonism of TMZ with Natural Compounds in Sports

Many athletes use natural extracts to enhance their athletic performance. The choice of these extracts is based on the supposed anabolic properties of the various natural compounds [61]. Aside from the naturally occurring substances that WADA prohibits (cocaine, cathinone, ephedrine, strychnine, and cannabinoids), there are still various classes of active principles that can have a variety of outcomes, eventually enhancing athletic performance. Dietary supplements that contain natural compounds are very popular among athletes, and there are some hypotheses why these compounds are combined with synthetic drugs. Some natural substances can have synergistic effects with trimetazidine. For example, saponins are a class of active principles that have amphiphilic properties. These compounds usually promote the absorption of other active substances [45]. Frequently used by athletes are the sterol saponins found in various plant products, such as *Tribulus terrestris*, *Discorea* sp., etc. [62,63].

Beetroot juice is also very popular among athletes due to its nitrate content. Following ingestion, anaerobic bacteria in the oral cavity use nitrate reductase to convert NO_3_ to nitrite (NO_2_), which is then converted to nitric oxide (NO) in the stomach. Numerous physiological processes that affect the use of oxygen during striated muscle contraction are triggered by NO. Hypoxic conditions facilitate the physiological mechanisms to reduce NO_2_, therefore NO (a vasodilator) is created in muscle regions that use or require more oxygen [64]. The nitrate-nitrite pathway has proven to have an important role in exercise efficiency [65]. In connection with TMZ, a synergistic effect may be expected between supplements containing nitrates and TMZ. Both categories are used in the management of angina pectoris. However, based on our current knowledge, there is a lack of studies that validate this synergistic effect.

Related to the TMZ mechanism of action that activates AMPK, facilitating glucose uptake, several natural compounds have been proven to have similar effects. Naringenin, a bioflavonoid with multiple effects and frequently encountered in dietary supplements used by athletes, activates AMPK and stimulates glucose uptake in skeletal muscle cells [66]. Also, diosgenin, a sterolic saponin, increases AMPK and acetyl-CoA carboxylase phosphorylation [67]. All these compounds could act in synergy when co-administered with TMZ.

An alternative hypothesis is that some natural compounds can antagonize the effects of TMZ. This is the case for all the compounds that stimulate fatty acid oxidation. In vitro and in vivo animal studies have demonstrated that resveratrol improves mitochondrial fatty acid beta-oxidation and acts in the opposite way compared to TMZ [68,69]. The same effect was noticed for some flavonoids [70].

However, more studies are needed to determine if these hypotheses are confirmed.

## 9. TMZ and Restrictive Diets in Sports

In the case of performance athletes, the diet occupies an extremely important place because it must ensure the energy and nutrients necessary during training, adaptation, and recovery between training sessions to have an optimal weight and to maintain their long-term health [71]. The impact of restrictive diets on sports performance is the subject of numerous studies, from the anti-inflammatory effect of the Mediterranean diet to the insulin-sensitizing effect of the high-fat diet [72,73]. A high-fat diet is often adopted by performance athletes not only for weight loss but also for the insulin-sensitizing effect. Studies show that the ketogenic diet (<50 g/day carbohydrate, >75% fat) could be beneficial both in increasing short-duration, vigorous-intensity endurance tests and in weight loss, which also enhances athletic performance [74]. A comparative study on the effect of TMZ and physical exercise in an animal model of insulin resistance shows common mechanisms by which serum glucose is reduced, glucose metabolism in the cell increases, and skeletal muscle insulin signaling-related protein ratios of p-IRS1/IRS1 and p-AKT/AKT increase. The mechanism of action is based on the nuclear factor erythroid 2 related factor 2 (Nrf2) signaling pathway, a main antioxidant signaling regulator that could prevent the development of metabolic syndrome and related cardiovascular diseases, as well as the inhibition by TMZ of the KAT enzyme [72]. Exercise can increase oxidative stress in an exercise-type and intensity-dependent manner, and the body’s ability to respond depends on each individual and the level of training [75]. TMZ, through upregulation of Nrf2/heme oxygenase-1 (HO-1) and downregulation of nuclear factor kappa B signaling, decreases the level of oxidative stress and apoptosis induced by exhaustive exercise [45]. Another study carried out in mice fed a high-fat diet shows that the expression of carbohydrate responsive element binding protein (ChREBP) and the de novo biosynthesis of fatty acids were reduced by decreasing the activity of enzymes like fatty acid synthase and acetyl CoA carboxylase in the high-fat diet with co-administration of the TMZ group compared with the diet group by increasing AMPK activity [76,77]. Studies show that TMZ alone or with exercise can improve mitochondrial quality in an obesity-induced skeletal muscle dysfunction characterized by mitochondrial dysfunction. A high-fat diet for 8 weeks induced obesity that was reduced by physical exercise, while TMZ did not affect body weight. Both physical exercise and TMZ prevented the accumulation of visceral fat when administering a high-fat diet [78].

## 10. Training, Competition, and “Fuel” Type? The Athlete’s Dilemma: Glucose or Fatty Acids?

The utilization of the phosphagen (PCr-ATP) system and anaerobic glycolysis plays a crucial role in distinguishing performance levels in specific sports such as weightlifting, bodybuilding, and sprinting (e.g., running 100 m). Therefore, the significance of muscle glycogen stores is highlighted, emphasizing the essential nature of a carbohydrate-rich diet and the importance of avoiding exhausting effort within 48 h before a competition to optimize athletic performance. During effort, the PCr-ATP system provides energy only for a few seconds, and although anaerobic glycolysis can be considered a “waste” of energy and lead to the formation of lactic acid as an end product, it has two major advantages: fast response and ATP formation in the absence of oxygen [79]. However, even during short-term intense efforts (of a few minutes), the release of energy is not necessarily sequential (PCr-ATP system, anaerobic glycolysis, aerobic glycolysis) [80].

In endurance efforts, things are more complicated. The balance between lipolysis and aerobic glycolysis can be adjusted according to different criteria, such as the quality of the “fuel” used, energy efficiency, oxygen consumption, the risk of oxidative stress, the type of diet (especially regarding carbohydrate intake and the ketogenic diet), and can be quantified using the respiratory quotient (RQ) as a parameter.

**Figure 5:**
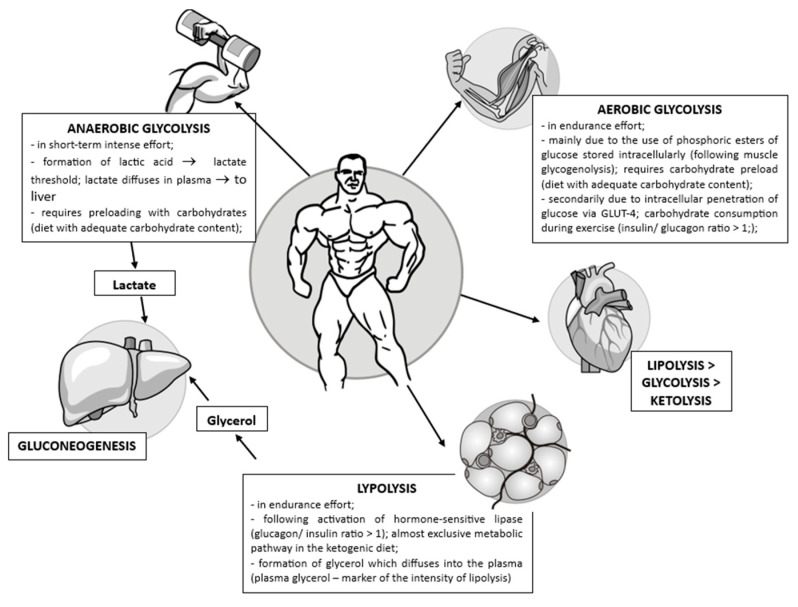
The use of different energy sources during endurance efforts.

The respiratory quotient (RQ) represents the ratio between the volume of CO_2_ released/Vol O_2_ consumed during respiration, a dimensionless number used to establish the basal metabolic rate. The RQ value varies from 1 in the case of aerobic use of carbohydrates, to 0.7 in the case of lipids, to 0.8 in the case of amino acids, and is 0 for the anaerobic use of glucose (see Figure 5) [81].

TMZ can induce metabolic flexibility to modulate the switch between energy substrates in ATP synthesis, as it increases intracellular glucose availability and decreases fatty acid oxidation; this is easily proven by measuring the RQ [82].

Preclinical studies on the rat heart performed at high altitude hypoxia with the combination of L-carnitine (a specific transporter of fatty acids in the mitochondria) and TMZ showed a clear improvement in cardiac metabolism by facilitating the beta-oxidation of fatty acids and promoting aerobic glycolysis [83]. On the other hand, clinical trials show that a balanced use of fatty acid beta-oxidation and glycolysis is essential for normal cardiac function, although the preferential use of glucose as an energy substrate has a favorable effect on myocardial ischemia [84].

On another note, the primary role of mitochondrial dysfunctions in age-related skeletal muscle loss (sarcopenia) is an important subject of consideration. Optimizing energy production via metabolic modulation could be an intriguing strategy to counteract mitochondrial dysfunctions and enhance stem cell function, thereby improving myogenesis during the aging process. Regarding the connection between myogenesis and oxidative metabolism, a study conducted on an experimental model of cachexia shows that reprogramming metabolism through 12 consecutive days of TMZ treatment induces the expression of myogenic genes in the skeletal muscles of mice, enhancing the levels of mitochondrial proteins and promoting oxidative metabolism in aging muscles [85]. Preclinical studies in mice show that it acts as an "exercise mimetic" and prevents sarcopenia and muscle dysfunction following cancer cachexia [86]. Therefore, TMZ enhances myoblast differentiation and fosters myogenesis, leading to the formation of new myofibers. This serves as an additional reason for its use as a performance-enhancing substance.

Additionally, Bucci et al. [87] postulate that human obesity is characterized by increased use of fatty acids by skeletal muscles concomitant with subcutaneous fat storage disorder, a phenomenon that leads to increased exposure of lean mass to oxidative stress. This maladaptation is effectively counteracted by TMZ.

We still do not have a final answer to the question of whether lipids or glucose are the most efficient energy substrate for sustained physical effort, but it is certain that TMZ as a metabolic modulator between lipo- and glycolysis improves sports performance, which is why TMZ is included on the prohibited list of WADA [7].

## 11. Conclusions

The mechanism of action of TMZ is complex, involving a metabolic modulation of lipid metabolism by inhibiting KAT while simultaneously activating biochemical pathways that favor the use of glucose for energy purposes. Key enzymes such as PDH or AMPK activated by TMZ connect glycolysis with mitochondrial metabolism, reducing the oxygen consumption of the cell. Even if this metabolic modulation does not bring substantial advantages in the case of occasional athletes, in the case of performance athletes, during competition, sometimes the difference is made in thousandths of a second. That is why TMZ is included by WADA on the list of substances prohibited for performance athletes during and outside the competition.

## Data Availability

Not applicable.
